# Mortality trends and sociodemographic factors associated with early death in sickle cell disease patients in the state of São Paulo

**DOI:** 10.1590/1984-0462/2024/42/2023113

**Published:** 2024-05-27

**Authors:** Nayara Dorta de Souza Avelino, Tulio Konstantyner, Kelsy Catherina Nema Areco, Juliana Moreira Franco, Josefina Aparecida Pellegrini Braga

**Affiliations:** a Universidade Federal de São Paulo, Escola Paulista de Medicina, São Paulo, SP, Brazil.

**Keywords:** Sickle cell disease, Mortality, Demographic data, Survival analysis, Development indicators, Death certificates, Doença falciforme, Mortalidade, Dados demográficos, Análise de sobrevida, Indicadores de desenvolvimento, Atestado de óbito

## Abstract

**Objective:**

To estimate trends in mortality rate and average age of death, and identify sociodemographic factors associated with early death in patients with sickle cell disease (SCD).

**Methods:**

An ecological and cross-sectional study was conducted using data from the Mortality Information System. All deaths of patients residing in the state of São Paulo from 1996 to 2015 with at least one International Disease Code for SCD in any field of the death certificate were included. Simple linear regression was used to estimate trends. The Log-rank test and multiple Cox regression were used to identify factors associated with early death.

**Results:**

The age-standardized mortality rate per million inhabitants increased by 0.080 per year (R^2^=0.761; p<0.001). When the events were stratified by age at death, the increase was 0.108 per year for those occurring at age 20 years or older, (R^2^=0.789; p<0.001) and 0.023 per year for those occurring before age 20 years old (R^2^=0.188; p=0.056). The average age at death increased by 0.617 years (7.4 months) per year (R^2^=0.835; p<0.001). Sociodemographic factors associated with early death identified were male gender (*hazard ratio* — HR=1.30), white race (HR=1.16), death occurring in the hospital (HR=1.29), and living in the Greater São Paulo (HR=1.13).

**Conclusions:**

The mortality rate and the average age of death in patients with SCD have increased over the last two decades. Sociodemographic factors such as gender, race, place of occurrence, and residence were found to be associated with early death.

## RESUMO

**Objetivo:**

Estimar as tendências da taxa de mortalidade e da idade média de morte e identificar os fatores sociodemográficos associados ao óbito precoce em pacientes com doença falciforme (DF).

**Métodos:**

Estudo ecológico e transversal realizado com dados do Sistema de Informações sobre Mortalidade. Foram incluídos todos os eventos de óbitos de pacientes residentes no estado de São Paulo de 1996 a 2015, que continham pelo menos um Código Internacional de Doenças para DF, em qualquer campo do atestado de óbito. As tendências foram estimadas por meio da regressão linear simples. Para a identificação dos fatores associados ao óbito precoce, foram realizadas análises de sobrevida, por meio da regressão de Cox simples e múltipla.

**Resultados:**

A taxa de mortalidade, padronizada pela idade, por milhão de habitantes, aumentou 0,080 ao ano (R²=0,761; p<0,001). Quando os eventos foram estratificados por idade do óbito, naqueles que ocorreram com 20 anos ou mais, o aumento foi de 0,108 ao ano (R²=0,789; p<0,001) e, nos que ocorreram antes de 20 anos, foi de 0,023 ao ano (R²=0,188; p=0,056). A idade média ao morrer aumentou 0,617 ano por ano (R²=0,835; p<0,001). Os fatores associados ao óbito precoce identificados no modelo múltiplo foram: sexo masculino (*hazard ratio* — HR=1,30), raça branca (HR=1,16), morte dentro do hospital (HR=1,29) e moradia na Grande São Paulo (HR=1,13).

**Conclusões:**

Houve aumento da taxa de mortalidade e da idade média de óbito com DF nas duas últimas décadas estudadas. Os fatores sociodemográficos sexo, raça, local de ocorrência e município de residência estiveram associados com a faixa etária do óbito.

## INTRODUCTION

Sickle cell disease (SCD) is an inherited hemolytic anemia, characterized by the production of an abnormal hemoglobin, specifically Hemoglobin S (HbS). It is the most common monogenic disease in Brazil and has a significant impact on public health.^1^ Its estimated incidence is one in every thousand births in Brazil, and one in every four thousand births in the state of São Paulo.^2,3^

Alves (1996) found that 78% of deaths among patients with SCD in Brazil occurred before the age of 29, and 37.5% of these deaths were in children younger than nine years old.^4^ In the 1970s, 30% of children born with SCD in the United States died before the age of five.^5^ However, survival rates for SCD patients have improved worldwide in recent decades due to early diagnosis through neonatal screening for hemoglobinopathies and advances in disease management and treatment.^5,6^

Studies on SCD in Brazil have identified varying mortality rates and average ages of death. These differences may be due to disparities in the healthcare systems across different states and the use of different estimation methods in each study.^3,7-9^ Despite recent nationwide research^10^ and studies in some Brazilian states,^1^ there is currently no published research on death related to SCD in the state of São Paulo, which is the most populous state in Brazil.

According to epidemiological studies, clinical characteristics, such as prematurity, nutritional status, and recurrent infections may contribute to poor survival rates in SCD patients.^9^ Additionally, sociodemographic factors, including sex, residence area, and health education have also been associated with the life expectancy in these patients.^3,11-13^ Health professionals should take these factors into consideration when treating SCD patients to improve care and increase survival rates. It is important to use representative samples in these studies to account for potential associations with lower survival.

The aim of this study was to estimate trends in mortality rate and average age of death over recent decades, as well as to identify sociodemographic factors associated with early death in patients with SCD residing in the state of São Paulo.

## METHOD

The present study had two designs: ecological and cross-sectional. The ecological design compared age-standardized mortality rates over time using aggregated data. The cross-sectional design examined factors associated with early death among subjects using individual data. Both designs were based on public data from the Mortality Information System (SIM), provided by the Ministry of Health of Brazil.^14^

All death events in the state of São Paulo that occurred between January 1^st^ 1996 and December 31^st^ 2015 were included, as per the availability of the SIM. We selected death records that contained at least one International Statistical Classification of Diseases and Related Health Problems (ICD) code for SCD in any field (basic cause, intermediate causes, immediate cause, and contributing causes). The following ICD codes were used to classify SCD: D57.0 (sickle cell anemia with crisis), D57.1 (sickle cell anemia without crisis), D57.2 (double heterozygous sickling disorders), D57.3 (sickle cell trait), and D57.8 (other sickle cell disorders).^15^

To deal with the effect of age, we calculated age-standardized mortality rates by adjusting for the age structure of the average population of residents in the state of São Paulo from 1996 to 2015, using data from the database of the Brazilian Ministry of Health (DATASUS).^16^ We considered two distinct age groups: pediatric (under 20 years old) and adult (20 years old or more). Following the World Health Organization (WHO) definition, we calculated annual age-standardized mortality rates by dividing the number of deaths of SCD patients in each age group by the corresponding age-specific population group living in the state of São Paulo, and then multiplying the result by one million. Therefore, the rate was expressed per million of age-compatible population (pmacp).^16^

Early death was defined as death occurring before the median life expectancy for SCD, which was 53.3 years for females and 56.5 years for males, according to a previous study on SCD mortality in Brazil.^9^

The study analyzed sociodemographic factors, including sex, race/color, place of death (hospital or non-hospital, such as another health facility, public road, or household), and residence area (living in the Greater São Paulo or in other cities within the state).

Furthermore, we analyzed the Municipal Human Development Index (MHDI) of the municipality where the residence was located. The MHDI is a composite measure that includes three indicators: opportunity to live a long and healthy life (longevity), knowledge access (education), and basic needs assurance (income). The MHDI was categorized dichotomously based on the median value in the state of São Paulo (MHDI=0.738).^17^

Continuous variables were presented as the mean and standard deviation (SD), while categorical variables were presented as absolute and relative frequencies. Trends in mortality rates and mean age at death over the years were estimated using simple linear regression, with the year as the independent variable and considering year 1996 as year zero. The assumptions regarding linear regression, such as the absence of serial autocorrelation, residuals distribution and homoscedasticity have been met.

A Kaplan-Meier curve represented the univariate associations with a comparison of curves using the log-rank test. To investigate factors associated with early death, a multivariate survival analysis was performed using Cox proportional hazards model. Only variables with a univariate association with a *p-*value < 0.20 were allowed to enter the multivariate analysis. All independent variables were included simultaneously using the "enter" method.^18^

Statistical analysis was performed using the Statistical Package for the Social Sciences (SPSS) version 20.0 (IBM Corp, NY, United States).^19^ Associations were considered statistically significant when p<0.05.

The research project received approval from the Research Ethics Committee of the Federal University of São Paulo (UNIFESP), under registration number 0057/2019, opinion number 3.138.864, and protocol number CAAE 06457519.0.0000.5505.

## RESULTS

From 1996 to 2015, there were 1,675 registered deaths of patients with SCD in the state of São Paulo. Of these, 876 (52.3%) were female and 799 (47.7%) were male. Out of all the patients, 1,625 (97%) had sickle cell anemia, 38 (2.2%) had double heterozygous or other sickle cell disorders, and 12 (0.8%) had sickle cell trait.

Of the total deaths, 35% were white, 32.4% were brown, 23.6% were black (396), 0.4% were Asian, and 0.1% were indigenous. Missing data accounted for 8.3% of the deaths. Among these deaths, 24.3% occurred before the age of 20. The average age at death was 32.0 years (SD=18.5), with females averaging 34.4 years (SD=19.0) and males averaging 29.4 years (SD=17.5).

**Figure 1:**
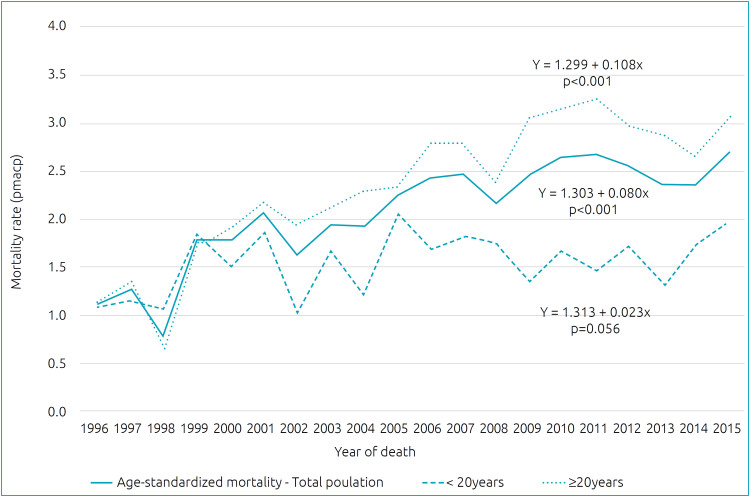
Temporal trend of age-standardized mortality rates per million of age-compatible population (pmacp) and age-specific groups of sickle cell disease patients’ deaths in São Paulo (1996–2015).

During the study period, the age-standardized mortality rate was 2.08 pmacp. The rate increased by 0.080 per year (R^2^=0.761; p<0.001). In the adult age group, the increase was 0.108 per year (R^2^=0.789; p<0.001), while in the pediatric age group it was 0.023 per year (R^2^=0.188; p=0.056) (Figure 1). The average age of death increased by 0.617 years (7.4 months) per year (R^2^=0.835; p<0.001).

Based on the adopted criteria, 88.4% of all deaths among SCD patients living in the state of São Paulo were classified as early deaths. The proportion of early deaths over the period decreased by 0.6 percentage points per year (R2=0.530; p<0.001).

**Figure 2:**
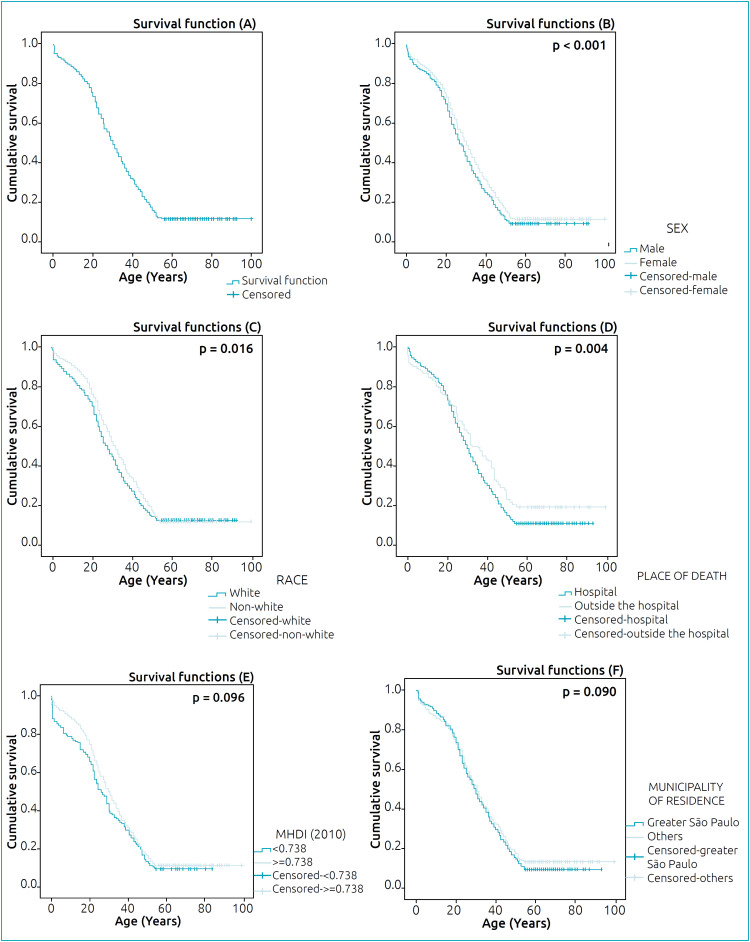
Cumulative survival functions for early death of subjects who died with sickle cell disease in the general population (A) and by subgroups of sex (B), race/color (C), place of death (D), MHDI - Municipal Human Development Index (E) and municipality of residence (F). São Paulo (1996–2015).

Figure 2A shows the overall survival over time. Male and white subjects died earlier when compared to females (p<0.001) and non-white subjects (p=0.016), as presented in Figure 2B and 2C.

Although Figure 2D shows a tendency for earlier deaths outside the hospital during the first two decades of life, death occurred earlier inside the hospital when all ages were analyzed (p=0.004). Subjects living in cities with lower MHDI tended to die earlier, with 13% of deaths in this group occurring in the first two years of life. However, there was no statistically significant difference when compared to those living in municipalities with a higher MHDI (p=0.096) (Figure 2E). In terms of place of residence, there was no statistically significant difference (p=0.090) between those living in Greater São Paulo and those living in other cities (Figure 2F).

The multivariate analysis identified four sociodemographic factors associated with early death: male sex (HR=1.30; p<0.001), white race (HR=1.16; p=0.007), and those dying in the hospital (HR=1.29; p=0.015) and living in the Greater São Paulo (HR=1.13; p=0.027). The MHDI was not significantly associated with early deaths in the multiple model (HR=1.18; p=0.062) (Table 1).

**Table 1:** Multiple Cox model of early death risk factors in sickle cell disease subjects. São Paulo (1996–2015).

Risk factor	Category	n	HR	95%CI	p-value
Sex	Male	799	1.30	1.17–1.45	<0.001
	Female	876	1.00		
Race/Skin color	White	587	1.16	1.04–1.30	0.007
	Non-white	949	1.00		
Place of death	Hospital	1542	1.29	1.05–1.58	0.015
	Out-of-hospital	133	1.00		
Municipality of residence	Grater São Paulo	918	1.13	1.01–1.26	0.027
	Other cities	756	1.00		
MHDI	<0.738	188	1.18	0.99–1.39	0.062
	≥0.738	1486	1.00		

HR: Hazard Ratio; CI: Confidence Interval; MHDI: Municipal Human Development Index.

## DISCUSSION

This study aimed to estimate the trends in the mortality rates and average age at death and to identify the sociodemographic factors associated with early death among subjects with SCD in the state of São Paulo between 1996 and 2015. Annually, the age-standardized mortality rate per million inhabitants increased by 0.080 and the average age at death increased by 0.617 years (7.4 months). Sociodemographic factors associated with early death identified were male gender, white race, death occurring in the hospital, and living in Greater São Paulo.

Over the studied period, the average age of SCD death in the state increased by 0.617 years per year. A study based on civil registry data between 2000 and 2018 showed an increase in the overall average life expectancy in the state of São Paulo by 3.2 months per year.^20^ The present study found a higher increase of 7.4 months per year in the mean age of SCD death. Although this may be a result of better living conditions in the general population, the greater increase in SCD patients’ rates suggests that advances in specific medical care for this group, including earlier diagnosis, follow-up, and better treatment of complications, have also played a role in increasing their life expectancy.^21^

Advancements in the clinical management of SCD have the potential to reduce mortality rates. However, the studied period showed an increase of 0.080 deaths per one million inhabitants per year. One possible explanation for this fact is the increased visibility of the disease, which is now identified early in life through neonatal screening for hemoglobinopathies in Brazil since 2001.^22^ As a result, more children and their families are being diagnosed with SCD, leading to more frequent registration of the disease on death certificates. Possible alternative explanations include changes in the epidemiological scenario over time, such as variations in disease incidence rates, regardless of improved diagnosis, and differences in access to health services.^23^

Our results are similar to recent studies on SCD mortality in the Brazilian population. These studies used data from SIM and estimated the mean age of death in males and females. Santo evaluated mortality and found mean ages of death of 29.4 and 33.3 years in males and females from 2000 to 2018, respectively.^10^ Mota et al. analyzed data from 1997–2017 and observed a higher incidence of deaths between the ages of 25 and 34 years.^24^

Even in another study conducted in Brazil that assessed hospital admission data and did not include out-of-hospital deaths, the mean age of death was found to be similar (33.5 years).^25^ Our study has identified an increase in the average age of death and mortality rate over the years, which is consistent with a similar study conducted in the United States from 1979 to 2005.^26^

Studies on SCD mortality have been conducted in various states of Brazil, but with specific age groups or using other data sources, such as hospital records.^7,8,25^ In the state of Maranhão, a comparison between two periods (pre- and post-neonatal hemoglobinopathies screening) resulted in an increase in the mortality rate,^7^ which corroborates our findings and reinforces the hypothesis of greater disease visibility after early screening for SCD.

Our study found that female mortality rates were highest, which is consistent with previous studies conducted in Bahia, Rio de Janeiro, and Minas Gerais.^3,8,9,11,25^ However, males had a higher frequency of earlier deaths. The reasons for these findings are unknown, but they may be related to cultural and behavioral factors.

Among SCD subjects who died during the study period, the mortality rate was 35% in white individuals, compared to 8.1% in the Bahia study, which used the same data source.^8^ This difference may be attributed to the heterogeneous ethnic distribution of the Brazilian population, which is characterized by racial diversity. In Bahia, specifically, most of the population is non-white.

Regarding death analysis, it was found that white subjects died earlier. This may be since the database primarily includes individuals born before the nationwide neonatal screening program was implemented. During that time, the disease was more commonly diagnosed in black people. Therefore, it is possible that the diagnosis of SCD in white patients only occurred when serious complications arose, resulting in earlier death.

In terms of occurrence location, early death was more frequent within the hospital. This is likely since the most severe patients usually seek or are referred to medical treatment at referral centers with greater hospital bed availability. This finding is supported by studies conducted in Minas Gerais and Bahia, which also found a higher prevalence of inpatient deaths in SCD patients.^3,8,11^ Moreover, our study found that less than 8% of patients died outside the hospital. These deaths may be related to other causes, such as external ones. This statement suggests that the patients died with SCD rather than from it.

When comparing places of residence, early death was found to be more frequent in Greater São Paulo than in other cities in the state. This probably occurred due to patient mobilization having clinical repercussions, as patients change their residence to be closer to tertiary hospitals and larger reference centers. These centers are mostly concentrated in the metropolitan region of the state. Similarly, studies conducted in Minas Gerais showed that approximately 78% of deaths occurred in individuals residing in urban areas.^3,11^

Although SCD subjects residing in cities with lower MHDI may have a higher risk of earlier death due to inadequate hospital infrastructure and lack of specialized services, this association was not statistically significant in the multiple models. We did not find any other national research that evaluated the impact of MHDI in SCD mortality.

It is noteworthy that the estimates and associations presented here are based solely on death records. Therefore, subjects with SCD who did not pass way were not included in the study. This precluded the estimation of survival rates or the identification of risk factors for earlier death, which can only be done by comparing living and deceased subjects. Moreover, during the review of death certificates, it was not possible to reliably determine if the cause of death was directly related to SCD. In other words, the patients had SCD at the time of their death, but it cannot be concluded that SCD was the direct cause of death. Furthermore, the investigation of factors associated with early death was limited to the sociodemographic factors available on the death certificate.

The patients analyzed may not be representative of all individuals with SCD who died during the study period. This is because the disease was only identified with greater sensitivity in Brazil after the implementation of neonatal screening in 2001 and the possible underreporting on death certificates due to diagnostic failures or incomplete information on the causes of death. Although the SIM has improved the accuracy of death certificates in recent decades in Brazil, there may still be inaccuracies.^27^

Still, this study is the first in Brazil to evaluate mortality related to SCD and factors associated with early death in the state of São Paulo. The methodological strategy involved searching for ICD codes in any field of death certificates, which potentially allowed for the identification of a wider range of SCD deaths.

We conclude that there was an increase in the mortality rate and the average age of death among patients with SCD over the 20-year period evaluated. Sociodemographic factors associated with early death included male sex, white race, in-hospital death, and living in Greater São Paulo. Although these factors are mostly not modifiable and should be interpreted with caution, knowledge of them can be useful for public policies and planning of health services aimed at monitoring patients with this hematological disease.

## Data Availability

The database that originated the article is available with the corresponding author.
